# Vessels in a *Rhododendron ferrugineum* (L.) population do not trace temperature anymore at the alpine shrubline

**DOI:** 10.3389/fpls.2022.1023384

**Published:** 2023-01-12

**Authors:** Silvia Piccinelli, Loïc Francon, Christophe Corona, Markus Stoffel, Lenka Slamova, Nicoletta Cannone

**Affiliations:** ^1^ Department Science and High Technology, Insubria University, Como, Italy; ^2^ Climate Change Impacts and Risks in the Anthropocene (C-CIA), Institute for Environmental Sciences, University of Geneva, Geneva, Switzerland; ^3^ Geolab, Université Clermont Auvergne, Centre National de la Recherche Scientifique (CNRS), Clermont-Ferrand, France; ^4^ Dendrolab.ch, Department of Earth Sciences, University of Geneva, Geneva, Switzerland; ^5^ Department of Forel for Environmental and Aquatic Sciences (F.A.), University of Geneva, Geneva, Switzerland; ^6^ Climate Change Research Centre, Insubria University, Como, Italy

**Keywords:** alpine shrub, dendroecology, wood anatomy, climate-growth relations, climatic signal loss

## Abstract

**Introduction:**

Mean xylem vessel or tracheid area have been demonstrated to represent powerful proxies to better understand the response of woody plants to changing climatic conditions. Yet, to date, this approach has rarely been applied to shrubs.

**Methods:**

Here, we developed a multidecadal, annually-resolved chronology of vessel sizes for Rhododendron ferrugineum shrubs sampled at the upper shrubline (2,550 m asl) on a north-facing, inactive rock glacier in the Italian Alps.

**Results and Discussion:**

Over the 1960-1989 period, the vessel size chronology shares 64% of common variability with summer temperatures, thus confirming the potential of wood anatomical analyses on shrubs to track past climate variability in alpine environments above treeline. The strong winter precipitation signal recorded in the chronology also confirms the negative effect of long-lasting snow cover on shrub growth. By contrast, the loss of a climate-growth relation signal since the 1990s for both temperature and precipitation, significantly stronger than the one found in radial growth, contrasts with findings in other QWA studies according to which stable correlations between series of anatomical features and climatic parameters have been reported. In a context of global warming, we hypothesize that this signal loss might be induced by winter droughts, late frost, or complex relations between increasing air temperatures, permafrost degradation, and its impacts on shrub growth. We recommend future studies to validate these hypotheses on monitored rock glaciers.

## 1 Introduction

In the European Alps, changes in land surface phenology (Asam et al., 2018; Xie et al., 2020), species richness (Cannone et al., 2007; Rixen et al., 2014; Lamprecht et al., 2018; Steinbauer et al., 2018; Malfasi and Cannone, 2020) or ecosystem productivity (Choler, 2015; Carlson et al., 2017; Filippa et al., 2019; Choler et al., 2021) have been described in studies relying on remote sensing time series or on resurveyed marked plots. As climate warming in the alpine region outpaces the global warming rate by 0.2 ± 0.1°C per decade (IPCC, 2014), these changes are widely perceived as the consequence of climate change. Yet, past land use and changes thereof have long-lasting legacy effects on ecosystems and their development (Tappeiner et al., 2021) as well, rendering an attribution of causes and effects sometimes difficult. In addition, vegetation dynamics at treeline have also been influenced by the constant decline of pastoralism and related human activities due to land abandonment since the Industrial Revolution (1850), making it challenging to disentangle the climate influence from direct human impacts or the absence thereof (Motta and Nola, 2001; Xie et al., 2018; Filippa et al., 2019; Lenoir et al., 2020). Directional cover changes, such as e.g. shrub expansion above the treeline, may thus erroneously be attributed to climate change when studying patterns of elevational range shifts for a large set of species (Forero-Medina et al., 2011; Guo et al., 2018). Besides, fine-scale species movements such as elevational range shifts could be largely constrained or confounded by local habitat availability (Guo et al., 2018). Consequently, identifying mechanisms behind vegetation and ecosystem changes – crucial to disentangle the respective impacts of climate, environmental, and anthropogenic factors on the observed dynamics – requires long-term, high-quality and continuous field-based monitoring time series, which are still extremely scarce, particularly in the alpine tundra biome (Le Moullec et al., 2019).

To date, shrub growth rings are the only proxy with annual resolution and exact dating control and thus have the potential to fill the above knowledge gaps retrospectively, thereby enhancing the understanding of dynamic ecosystem processes that occur naturally or are driven by anthropogenic activities (e.g., Seidl et al., 2017) in this remote biome. Site-specific relationships between climate variables and shrub radial growth have thus been assessed with dendroecological techniques (Myers-Smith et al., 2015b) and were proven particularly valuable to improve our understanding of the impacts of global warming on plant productivity over multidecadal timescales. In the Alps, above the treeline, several dendroecological studies thus demonstrated a positive effect of longer growing seasons and increased summer temperatures on shrub ring widths (Carrer et al., 2019; Francon et al., 2021).

Although ring width (RW) is an easy-to-measure parameter and widely used in dendrochronological research (e.g., Grissino-Mayer and Fritts, 1997), it integrates considerable inherent non-climatic information, such as age and size trends (Cook and Kairiukstis, 1990; Weiner and Thomas, 2001; García-González et al., 2016), biological memory effects (Fritts, 1976; Esper et al., 2015), external disturbances (Rydval et al., 2018) or simply unexplained variability (Cook, 1985). Moreover, RW shows an integrated response to climate conditions in the previous and the current year (Fritts, 2001), rendering an interpretation of climate-growth relationships challenging (Kagawa et al., 2006). Finally, since the mid- to late-20^th^ century, temperature-sensitive trees (D’Arrigo et al., 2004; Wilmking, 2005; Büntgen et al., 2008; D’Arrigo et al., 2008; Oberhuber et al., 2008; Leonelli et al., 2009) and shrubs (Buchwal et al., 2020; Francon et al., 2021) from the Arctic and Alpine regions were shown to exhibit complex and non-linear radial growth responses to warming climate. This phenomenon is known as the “divergence problem” and implies that radial growth becomes increasingly decorrelated from temperature in a warmer world (Briffa et al., 1998; Driscoll et al., 2005; D’Arrigo et al., 2008).

To overcome the above-mentioned limitations, the range of parameters characterizing annual rings and their use as environmental proxies has notably been diversified during recent decades. In particular, the measurement of maximum latewood density (MXD), determined by the size of cells and the thickness of their walls, plays a significant role in late Holocene paleoclimatology (Briffa et al., 2004; Björklund et al., 2019) as it is, for conifer species growing in cooler high-latitude and high-elevation environments, more strongly coupled to growing season air temperature than RW alone (Schweingruber et al., 1978; Björklund et al., 2019). Over the last years, the development of fully- and semi-automated processing approaches of high-resolution imagery now allows delving into the microscopic component of xylem trait features and to relate cell characteristics to environmental signals (von Arx and Carrer, 2014; Ziaco et al., 2016; Björklund et al., 2020; Lopez-Saez et al., 2023). The annual variability of xylem cell traits relies on mechanistic processes linked to the plant metabolism and reveals consequences of climate limitations on its growth (Wegner et al., 2013). In other words, the mechanistic link between climate and growth response is simplified using the cell characteristics with their direct structure-function link of xylem cells (Hacke et al., 2015; Björklund et al., 2020).

Yet, to date, the discipline, known as quantitative wood anatomy (QWA), has rarely been used on dwarf shrubs and, at best, over short time periods. For instance, focusing on a 6-year period of *Vaccinium myrtillus* at an experimental site in the European Alps, Anadon-Rosell et al. (2018) showed a reduction of vessel size as a response to experimental CO_2_ enrichment and soil warming. Similarly, Nielsen et al. (2017) focused on a ten-year window (2001-2011) to show a positive link between vessel size and radial wood increments of *Betula nana* in Greenland, whereas Lehejček et al. (2017) demonstrated the potential of *Juniperus communis* shrub anatomical parameters to trace past summer temperature and standardized precipitation evapotranspiration index (SPEI) fluctuations in the Arctic.

In this study, we compare the climatic signal of ring width and xylem anatomical trait chronologies of *Rhododendron ferrugineum* sampled above treeline (2550 m asl) on a north-facing inactive rock glacier in the Italian Alps to test the potential of QWA approaches in shrub dendroecology. The objective of our study is twofold: (1) on the one hand, we aim at gaining further insights into the climatic drivers of xylem anatomy of an alpine shrub species. To this end, we hypothesize that the climatic signals may differ and might be exacerbated in time-series of wood-anatomical variables compared to ring width chronologies. (2) On the other hand, we aim at comparing the response of RW and xylem anatomy to recent global warming. Here, we hypothesize that shrub RW and anatomical traits have been affected by the relaxation of the limiting conditions observed at high elevation sites, presumably reflecting the divergent growth response reported since the late 20^th^ century to recent climate warming (e.g., Briffa et al., 1998). Hence, in our study, *R. ferrugineum* growth is expected to suffer from a loss in climate sensitivity, which might even be enhanced in xylem cells as they are directly subjected to climate limitations (Björklund et al., 2020).

## 2 Material and methods

### 2.1 Study site

**Figure 1:**
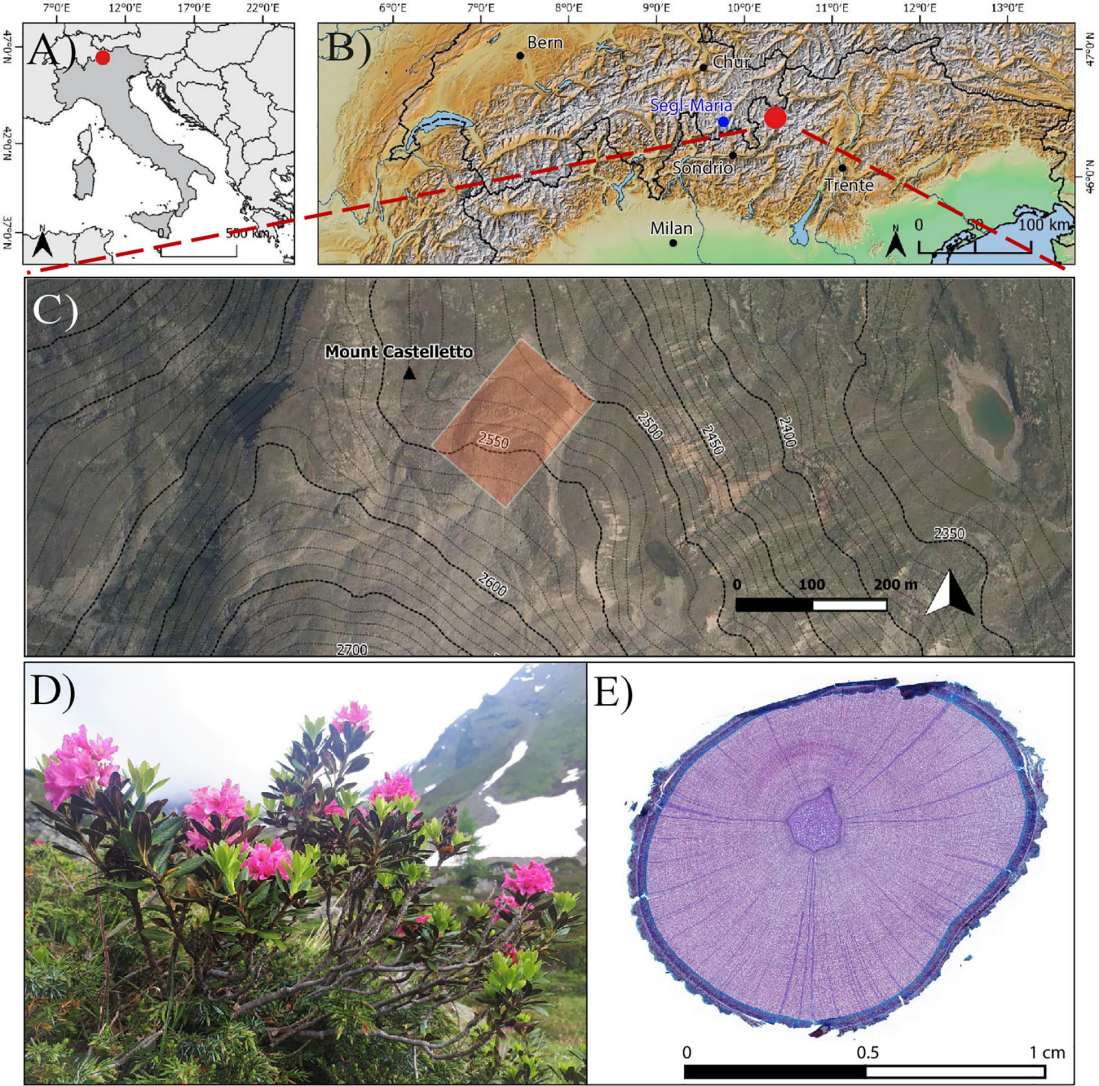
**(A–C)** Location of the study site in the Central Italian Alps, Upper Valtellina region **(B)**. The study focused on **(D)** *Rhododendron ferrugineum* specimens growing on the Castelletto rock glacier from which **(E)** stem micro-sections were prepared and analyzed.

The study was conducted on the north-facing Castelletto rock glacier (46°29’56’’N, 10°11’26’’E, **Figures 1A-C**) in the Upper Valtellina valley, Italian Alps (Calderoni et al., 1998). The region is part of the Austroalpine domain (Froitzheim et al., 2008) and is situated between the Periadriatic and the Engadine tectonic lines (Peña Reyes et al., 2015). Geomorphic surveys of the Castelletto rock glacier indicate a history comprising a glacial expansion during the Late Glacial overprinted by subsequent periglacial and slope processes (Guglielmin et al., 1994; Calderoni et al., 1998) which then led to the deposition of slope materials and rock glacier development during the Holocene (Calderoni et al., 1998; Guglielmin et al., 2004). Periglacial features at the site include avalanche cones as well as several features typical for periglacial environments (i.e. polygons and stripes, gelifluction lobes, and earth hummocks) (Calderoni et al., 1998).

The rock glacier is currently composed of an active (>2580 m asl) and an inactive (2500-2580 m asl) part (Guglielmin, 1989; Calderoni et al., 1998; Guglielmin and Smiraglia, 1998). Active features - consisting of two lobes (Guglielmin, 1989; Calderoni et al., 1998) - are covered by a limited and scattered cover of pioneer herbaceous species that are well adapted to mechanical disturbances (Cannone and Piccinelli, 2021). On the inactive part - characterized by an elongated, lobe-shaped feature spreading downvalley (Guglielmin, 1989; Calderoni et al., 1998) - vegetation consists of alpine dwarf shrub communities, including *Rhodoretum-Vaccinietum, Loiseleurieto-Cetrarietum* and *Junipero-Arctostaphyletum* encroaching the alpine grassland ecotone, dominated by *Caricetum curvulae, Curvulo-Nardetum*, *Festucetum variae*, *Nardetum alpigenum*, *Aveno Nardetum* associations (Cannone and Piccinelli, 2021).

Meteorological data do not exist at the study site. Yet, according to the time series from the automatic weather station Segl-Maria (46°26’ N, 9°46’ E, 1804 m asl), located 33 km to the W of the study site, mean annual air temperatures and precipitation totals are 2.15 ± 0.67°C and 997 ± 206 mm, respectively, over the period 1960-2019.

### 2.2 Sampling strategy

In this study, we sampled *Rhododendron ferrugineum* L. (Ericaceae) (**Figure 1D**), an evergreen dominant shrub species in the subalpine belts of non-calcareous alpine regions (Pornon and Doche, 1996; Căprar et al., 2014; Charrier et al., 2014), from the inactive part of the Castelletto rock glacier at elevations comprised between 2540 and 2580 m asl. Another motivation for the species selection – in addition to its wide distribution – is the potential of *R. ferrugineum* for dendroecological analyses as recently stressed by several studies (Francon et al., 2017; Francon et al., 2020a; Francon et al., 2020b; Francon et al., 2021) as well as the longevity and clearly identifiable annual rings of the species. We selected a total of 14 isolated individuals, separated from each other by at least 4 m, to avoid replication from the same individual. For each individual, we cut two to three cross-sections (42 sections in total), from the root collar to the apex of the main stem, at an interval of 5-10 cm between each cross-section, with the aim to realize a serial sectioning approach (Kolishchuk, 1990). Identification of the root collar was possible due to the limited size of individuals (crown size within 1 m diameter) and a careful sampling involving soil excavation up to 10 cm under the surface (Malfasi and Cannone, 2020). For each cross-section, we prepared a 20-μm thick micro section using a Leica Rotary Microtome. Micro-sections were stained with safranin (1% w/v in 70% v/v ethanol) and Astra blue (1% w/v in 100% ethanol) to enhance ring boundaries (**Figure 1E**) and permanently fixed with Euparal on microslides following standard preparation procedures (Schweingruber et al., 2011; Gärtner et al., 2015; von Arx et al., 2016).

### 2.3 Ring-width and anatomical trait measurements

We carefully inspected each cross-section to detect wedging rings and measured RW along three radii from high-resolution (1200 dpi), digitized images using the CooRecorder 9.0 software. We first developed a RW chronology following a three-step procedure which included the cross-dating and averaging of (1) three radii within each cross-section, (2) three cross-sections of the same individual, and (3) RW series from the 14 individuals (Francon et al., 2017; Francon et al., 2020a; Francon et al., 2020b; Francon et al., 2021).

Based on image quality, series length and correlation with the master chronology (r > 0.6), we selected 9 individuals for QWA analyses (Fonti et al., 2013). The sample size is in line with recent studies investigating dwarf shrub anatomical traits and water potential (5 individuals; Anadon-Rosell et al., 2018; Ganthaler and Mayr, 2021), as well as tree species anatomical parameters, including *Larix siberica* Ldb. (5 individuals; Fonti et al., 2013), *Pinus sylvestris* L. (8-9; Pritzkow et al., 2014; Seo et al., 2014), *Picea abies* (L.) Karst. (8 individuals; Castagneri et al., 2015), *Larix decidua* Mill. and *Pinus cembra* L. (6-7 individuals; Carrer et al., 2017). For each individual, we performed anatomical measurements along two radial subareas of the root collar cross-section (2-mm wide bands with lengths extending from bark to pith, selected with the Zeiss ZEN software), while carefully avoiding incomplete ring sequences, rotten parts, callus tissue, reaction wood, or mechanical damage (Carrer et al., 2017). Using the ROXAS 3.0.586 image analysis software (von Arx and Carrer, 2014), we measured eight vessel-related anatomical traits within each ring: number of cells (CNo), mean vessel lumen area (MLA), the 25^th^ and 95^th^ percentiles of lumen area distributions (MLA25 and MLA95), hydraulically weighted mean vessel diameter (Dh), theoretical total (Kh) and xylem-specific (Ks) hydraulic conductivities as well as cell density (CD) (Anadon-Rosell et al., 2018). The ROXAS software (von Arx and Carrer, 2014) allowed thorough analysis of anatomical features by coupling automated image-analysis tools, with accurate manual editing at both ring (e.g., digitizing ring boundaries not automatically recognized) and cell (e.g., correction of anomalous structures) levels. After applying proper image calibration and sample-specific configurations, the anatomical traits were detected using an automated segmentation process, improved by manual editing (von Arx et al., 2016, Garcia-Pedrero et al., 2018).

### 2.4 Chronology development and variable selection

**Figure 2:**
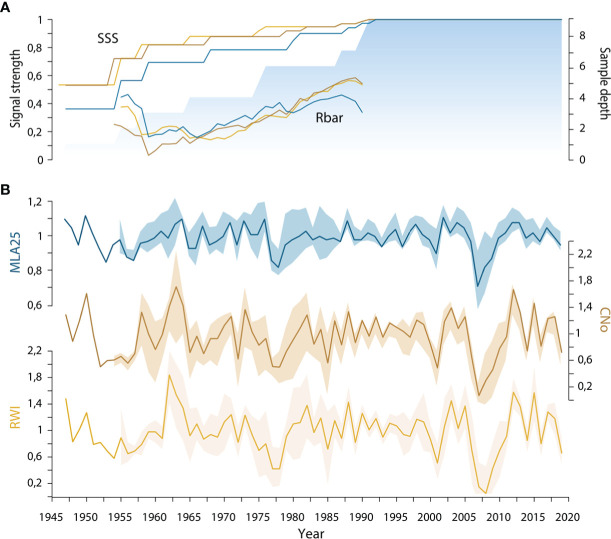
**(A)** Running detrended signal strength of the three selected *R. ferrugineum* chronologies (blue = MLA25, beige = CNo, yellow = RW) including mean inter-series correlation (rbar) and subsample signal strength (SSS) calculated at the first year of the moving window (30-year time window). The blue area indicates the sample depth; **(B)** Standard chronologies of *R. ferrugineum* with ribbons designating parameter indices ± standard deviation. RW= ring width, CNo= cell number, and MLA25= the 25^th^ percentiles of lumen area distribution.

Using the dplR package (Bunn, 2008) for R software (R Core Team, 2016), we detrended individual series of vessel anatomical traits using a cubic smoothing spline with a 50% frequency response at 30 years (Cook and Peters, 1981; Cook, 1987) to eliminate non-climatic trends (e.g., age-related growth trends and/or effects of natural or human-induced disturbances) and to maximize interannual variations (Francon et al., 2017). The resulting growth indices were averaged in annually-resolved chronologies using a bi-weight robust mean designed to reduce the influence of outliers (Cook and Peters, 1981). We assessed the robustness of each chronology by computing the mean inter-series correlation (rbar), the expressed population signal (EPS; Wigley et al., 1984), and the subsample signal strength (SSS; Wigley et al., 1984; Buras, 2017) (**Table 1**). Accordingly, all further analyses refer to the minimum period fulfilling the chronology coherence requirements (1960-2019). Kh and CD chronologies which did not reach the commonly accepted 0.85 threshold were disregarded. As many of the vessel-related variables are correlated and carry redundant information (García-González et al., 2016), we used principal component analysis (varimax-rotated PCA) to reduce the full suite of variables to a subset of different, statistically meaningful variables by using the chronologies calculated from the detrended individual time series (Akhmetzyanov et al., 2019) (**Figure 2B**). The selection was based on the ordination of variables according to the first two principal components (PCs).

**Table 1:** Characteristics of *Rhododendron ferrugineum* chronologies including all wood parameters (RW= ring width; CNo= cell number; MLA= vessel lumen area; MLA25 = 25^th^ percentile of lumen area distribution; CA95= vessel lumen area at 95^th^ percentile; DH= hydraulically weighted mean vessel diameter; Kh= theoretical total hydraulic conductivity; Ks= theoretical xylem-specific hydraulic conductivity; CD= Cell density): mean annual size (± standard deviation) computed on raw chronologies and signal strength (rbar and SSS), first-order autocorrelation (AR1) and intercorrelation (Intercorr) calculated after detrending (standardized – spline y=30 f=0.5) over to the entire period (1946-2019).

N° indiv	First year	Last year	Mean age		Age range	
9	1946	2019	49		74	
Parameter	Raw		Standardized – spline y=30 f=0.5			
	Units	Size	rbar	SSS	AR1	Intercorr
RW	mm	0.13 ± 0.05	0.483	0.889	0.266 ± 0.18	0.607 ± 0.14
CNo	no.	388 ± 212.23	0.484	0.887	0.236 ± 0.18	0.614 ± 0.15
MLA	μm²	151 ± 32.02	0.332	0.838	0.183 ± 0.18	0.457 ± 0.18
MLA25	μm²	153 ± 31.30	0.288	0.819	0.225 ± 0.19	0.395 ± 0.18
CA95	μm²	155 ± 31.72	0.278	0.822	0.215 ± 0.19	0.378 ± 0.15
DH	μm	15.4 ± 2.24	0.331	0.841	0.182 ± 0.20	0.375 ± 0.20
Kh	m^4^*s^-1^*MPa^-1^	4.17E^-10^ ± 3.38E^-10^	0.026	0.679	-0.024 ± 0.12	0.059 ± 0.17
Ks	m^2^*s^-1^* MPa^-1^	0.0011 ± 0.0005	0.311	0.833	0.075 ± 0.22	0.447 ± 0.16
CD	no./mm²	1168 ± 219.15	0.021	0.672	0.154 ± 0.21	-0.104 ± 0.26

All the chronologies share the same sample size, chronology length, mean age, and age range.

### 2.5 Climate-growth relationships

For the analysis of climate-growth relationships, we used meteorological data from the MeteoSwiss automated weather station Segl-Maria (SIA; 46°26’ N, 9°46’ E). To assess potential shifts in shrub growth responses to climate, we investigated the entire period covered by meteorological records (1960-2019) as well as two subperiods (1960-1989 and 1990-2019) of sufficient length (i.e. 30 years) to be considered as climate normal. The two subperiods were chosen according to the results obtained by computing moving correlation functions (MCFs) over a 30-year time window to test stability over time (see below).

We performed monthly and seasonal bootstrapped correlation functions (BCFs) and 30-year moving correlation functions (MCFs) using the Treeclim package (Zang and Biondi, 2015) for R software. To this end, we detrended meteorological data prior to climate-growth relationship analyses using the standardization procedure employed for the series of anatomical traits. The time window considered for BCFs and MCFs spans from August of the year preceding growth-ring formation (n−1) to current August (n). In addition, to retrieve more detailed information on anatomical and radial growth responses to climatic parameters (Jevšenak and Levanič, 2018; Thomte et al., 2020), we computed MCFs between RW and those vessel-related trait series that were identified from the PCA (i.e. CNO and MLA25, see below) and daily temperature and precipitation series from the Segl-Maria weather station averaged over time periods ranging from 10 to 150 days data using the dendroTools R package (Jevšenak and Levanič, 2018).

## 3 Results and discussion

### 3.1 Sample characteristics and selection of complementary variables

On average, the nine individuals included in our chronologies had an age of 49 years, with mean ring widths and mean lumen areas of 0.13 mm and 151 μm², respectively (**Table 1**). The Varimax PCA (**Figure S1**) realized on all parameters measured on the nine individuals discriminates two clusters of parameters. The first cluster includes variables related to cambium cell division, RW and CNo, whereas the second cluster is composed of variables related to cell enlargement (MLA, MLA25, MLA95, Dh, Ks). A similar dichotomy has been reported in published work for various tree (Wang et al., 2002; Vaganov et al., 2006; Martin-Benito et al., 2013; Pritzkow et al., 2014; Carrer et al., 2017) and shrub (Olano et al., 2012; Lehejček et al., 2017; Anadon-Rosell et al., 2018) species. Based on the loadings of variables on the first two principal components, we considered the RW, CNo, and MLA25 chronologies for further analysis (**Figure S1**). In addition, the vicinity between CNo and RW chronologies in the PCA biplot (**Figure S1**) confirms that radial growth is more strongly driven by cell division than by cell enlargement (Panyushkina et al., 2003; Martin-Benito et al., 2013; Pritzkow et al., 2014). Similarly, the positive loadings of the three variables on PC1 and PC2 suggest a positive pathway between RW/CNo and MLA25. We hypothesize that in the absence of drought or frost, wider vessels will be beneficial for increased water conductivity which in turn has benefits for radial growth (Tyree et al., 1994). Similar results have been previously reported in tundra shrubs – such as *Juniperus communis* (Lehejček et al., 2017) and *Betula nana* (Nielsen et al., 2017) –, forbs (Olano et al., 2013), or conifer species (Fonti et al., 2013; Pritzkow et al., 2014; Ziaco et al., 2014).

We also show that over the period 1946-2019, inter-series correlation statistics (i.e. SSS and rbar) are higher in the RW and CNo detrended chronologies (rbar = 0.48 and SSS = 0.89 for both chronologies) than in the MLA25 detrended chronology (rbar=0.29, SSS=0.82, **Table 1**, **Figure 2A**). At the same time, the moving rbar, computed over 30-year time windows, steadily increased from 0.22 (1960-1989) to 0.45 (1990-2019) in the MLA25, from 0.38 to 0.60 in the CNo, and from 0.42 to 0.54 in the RW chronologies, respectively (**Table S1**). The first-order autocorrelation (AR1) –0.27 for RW, 0.24 for CNo, and 0.23 for MLA25 (**Table 1**) – is comparable between the three detrended chronologies. One may also realize that the inter-series correlations computed for the MLA, MLA25, and CNo chronologies in this study are (considerably) higher than in most other studies (e.g., Lehejček et al. (2017) for *J. communis* Arctic dwarf shrubs, Carrer et al. (2017) for high-elevation *L. decidua* and *P. abies* trees in the Alps, or Seo et al. (2012) and Björklund et al. (2020) for *P. sylvestris* in subarctic regions) where the rbar only rarely reached a value of 0.3.

Yet, the shared variance explained by our *R. ferrugineum* wood anatomical chronologies remains lower than in the RW chronology. Similarly, weak empirical signal strength has been reported for wood anatomical chronologies built from tree species (e.g., *Quercus* sp. (Tardif and Conciatori, 2006; Fonti and García-González, 2008) or *Castanea sativa* (Fonti and García-González, 2004)). In their case, the weaker signal has been attributed to lower year-to-year variability in vessel lumen area chronologies as compared to RW and CNo chronologies (Olano et al., 2012; Liang et al., 2013; Martin-Benito et al., 2013; Pritzkow et al., 2014; Ziaco et al., 2016) and to higher inter-individual variability in vessel traits than in RW (Garcia-Cervigon et al., 2021).

The fairly robust metrics that we computed for shrub anatomical chronologies are very encouraging because shrubs usually exhibit greater inter-individual heterogeneity than trees (Lehejček et al., 2017; Buras et al., 2017). We, therefore, posit that the statistics that we find in the case of *R. ferrugineum* do not only validate the accuracy and representativeness of our measurements and the cross-dating approach (Carrer et al., 2017) but that they presumably point to a common macro-environmental (*i.e*. climatic) influence on growth as well (Liang and Eckstein, 2009). Nevertheless, although common variance may well be explained by common climate forcing (Wigley et al., 1984), for various species, robust climatic signals have been extracted from anatomical chronologies with low year-to-year common signals (Yasue et al., 2000; Campelo et al., 2010; Olano et al., 2013; García-González et al., 2016; Carrer et al., 2017). Furthermore, one should keep in mind that the strength of the common signal cannot be interpreted solely in climatic terms because common variance may also arise from other factors (e.g., pests and disease, soil and air contamination) (Wigley et al., 1984).

### 3.2 Climatic drivers of ring width and cellular chronologies over the 1960-1989 period

Over the 1960-2019 period, bootstrap correlation functions (BCFs) computed between RW, CNo, and MLA25 chronologies and monthly climate data from Segl-Maria show comparable profiles (**Figure S2**). However, moving correlation functions (MCFs) computed over a 30-year time window demonstrate that these relations are not stable over time and strongly differ for the 1960-1989 and 1990-2019 periods (**Figure S3**). Therefore, we decided to examine both subperiods successively.

**Figure 3:**
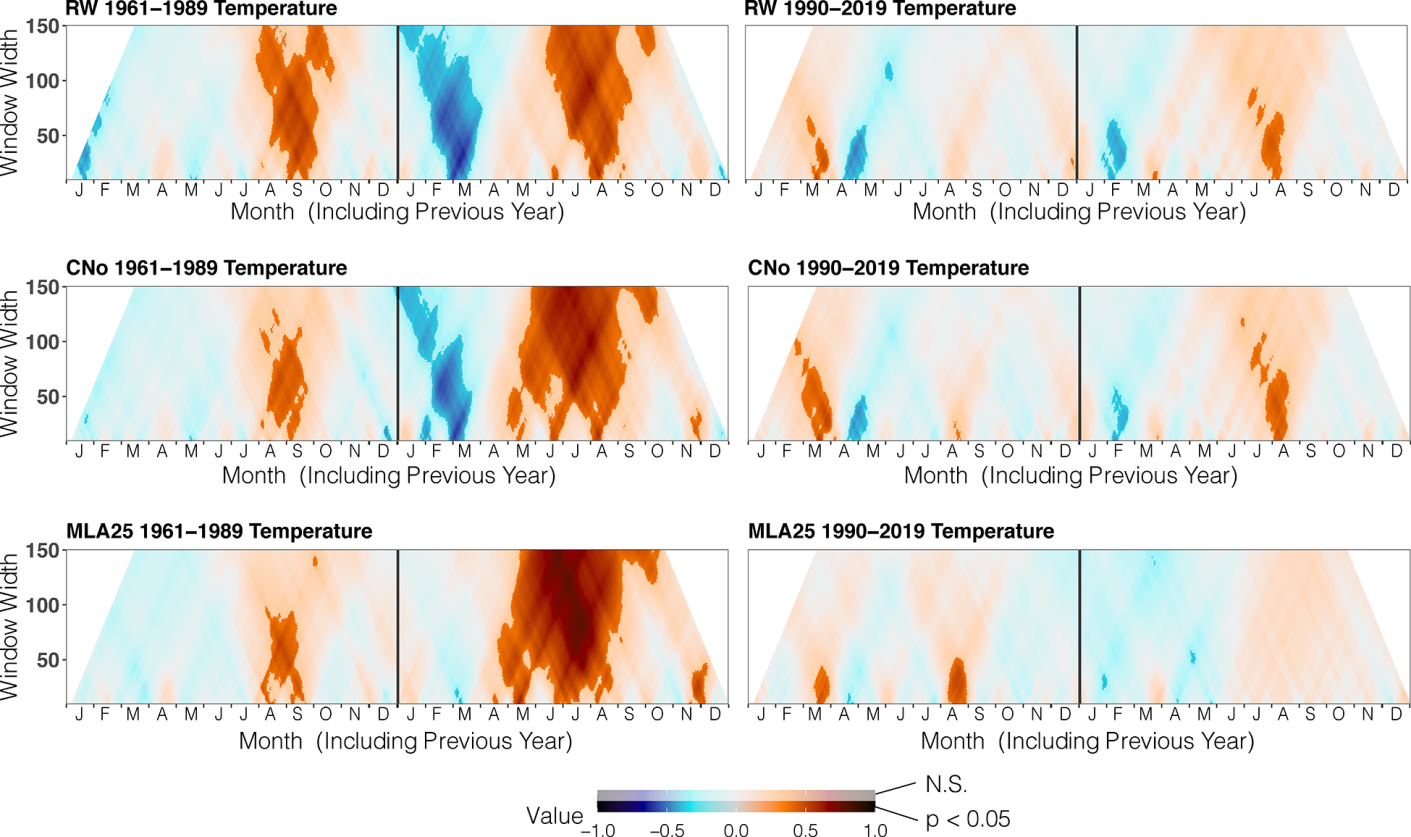
Correlation between the three selected chronologies (RW= ring width, CNo= cell number, and MLA25 = 25^th^ percentile of lumen area distribution) with daily temperature data over time periods (1960-1989, 1990-2019), ranging from 10 to 150 days data (window width), using the dendroTools R package. In the graphs, R2 values are shown, with significant values (p < 0.5) displayed with brighter colors than not significant ones (N.S., p > 0.05).

**Figure 4:**
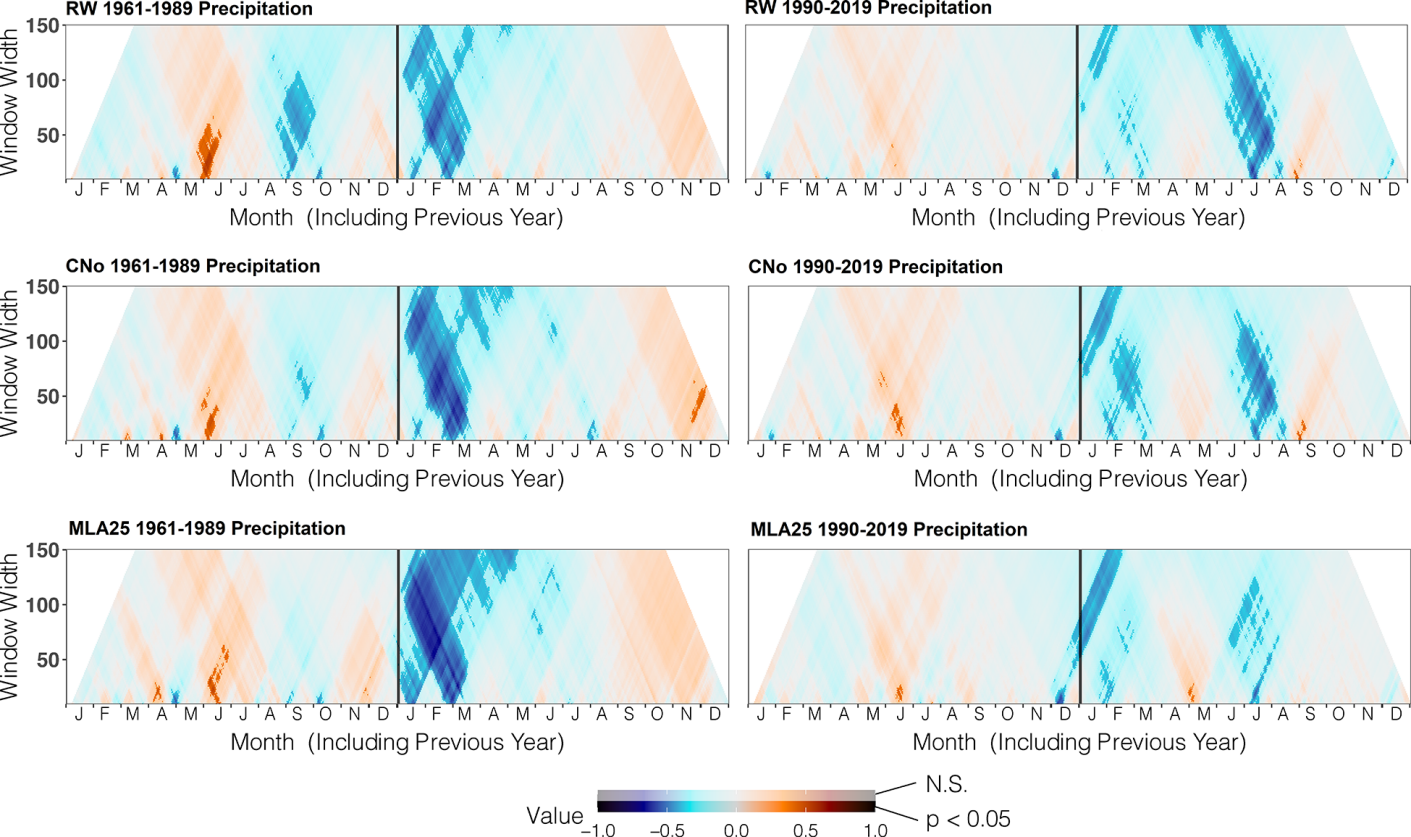
Correlation between the three selected chronologies (RW= ring width, CNo= cell number, and MLA25 = 25^th^ percentile of lumen area distribution) and daily precipitation data from the automated weather station Segl-Maria over time periods (1960-1989, 1990-2019), ranging from 10 to 150 days data (window width), using the dendroTools R package. In the graphs, R2 values are shown, with significant values (p < 0.5) displayed with brighter colors than not significant ones (N.S., p > 0.05).

Over the 1960-1989 period, the RW and CNo chronologies show the largest correlations with spring and summer air temperatures – averaged over a time window extending from June 10 to September 17. These temperatures explain 42% (r=0.646, p<0.05) and 47% (r=0.683, p<0.05) of the variability of RW and CNo, respectively (**Figure 3**; **Table 2**). RW (r=0.557, p<0.05) and, to a lesser extent, CNo (r=0.490, p<0.05) are also affected by temperature in August in the year preceding ring formation (n–1). The RW and CNo chronologies also show an inverse relationship with winter temperatures averaged over February 24 – March 21 (r= -0.630, p<0.05) and February 24 – March 10 (r= -0.591, p<0.05), respectively (**Figure 3**; **Table 2**). In terms of precipitation, snowy winters (January 13 – March 4, **Figure 4**; **Table 2**) had a negative effect on RW and CNo.

**Table 2:** Highest correlation coefficient values occurring in specific time periods calculated between the three selected chronologies (RW= ring width, CNo= cell number, and MLA25 = 25th percentile of lumen area distribution) and daily climatic data (temperature and precipitation).

TEMPERATURE				
	1960-1989		1990-2019	
	R-value (optimum window)	Period	R-value (optimum window)	Period
Current summer				
MLA25	0.801 (121)	May 08 – Sept 05	NS	NS
CNo	0.683 (99)	June 11 – Sept 17	0.493 (38)	July 19 – Aug 25
RW	0.646 (98)	June 10 – Sept 15	0.476 (46)	July 09 – Aug 23
Previous* late summer				
MLA25	0.528 (66)	July 22* – Sept 25*	0.521 (38)	Aug 01* – Sept 07*
CNo	0.490 (67)	July 21* – Sept 25*	NS	NS
RW	0.557 (89)	July 22* – Oct 18*	NS	NS
Current winter				
MLA25	-0.397 (18)	Feb 24 – March 13	-0.418 (29)	Jan 12 – Feb 09
CNo	-0.591 (15)	Feb 24 – March 10	-0.410 (23)	Feb 03 – Feb 25
RW	-0.630 (26)	Feb 24 – March 21	-0.440 (47)	Jan 19 – Mar 06
PRECIPITATION				
	1960-1989		1990-2019	
	R-value (optimum window)	Period	R-value optimum window	Period
Current winter				
MLA25	-0.684 (67)	Jan 12 – March 19	-0.492 (75)	Dec 3* - Feb 14
CNo	-0.638 (38)	Feb 11 – March 20	-0.505 (22)	Jan 23 – Feb 13
RW	-0.598 (51)	Jan 12 – March 13	-0.434 (22)	Jan 23 – Feb 13
Current summer				
MLA25	NS	NS	-0.548 (21)	July 01 – July 21
CNo	NS	NS	-0.505 (22)	July 02 – July 21
RW	NS	NS	-0.601 (17)	July 03 – July 19

*=Previous year; NS, Not Significant.

The positive influence of growing season temperatures on shrub radial growth has been reported in many dendroecological studies, from both arctic and alpine sites across the Northern Hemisphere (Myers-Smith et al., 2015a), including the Alps (Francon et al., 2017) and the Himalayas (Liang and Eckstein, 2009; Li et al., 2013; Lu et al., 2015). Regarding the negative association between *R. ferrugineum* ring widths and winter precipitation detected at our site, it seems likely that a more persistent snowpack in spring/early summer will shorten growing season length, and ultimately, limit shrub growth (Francon et al., 2017; Francon et al., 2020b; Pellizzari et al., 2014; Carrer et al., 2019). Finally, warm temperatures during the summer and fall preceding ring formation (n–1) positively influence (1) mycorrhizal activity by maintaining soils above freezing (Oberhuber, 2004; Oberhuber et al., 2008), (2) carbohydrates accumulation (Hoch et al., 2003; Francon et al., 2017) and (3) cambial zone formation (Vaganov et al., 2006; Martin-Benito et al., 2013). On the other hand, it is less obvious to find clear explanations for the negative association between winter temperature and the RW/CNo chronologies. We hypothesize that warm temperatures during winter may reduce snowpack depth, modify insulating properties (Domine et al., 2016), and may thus favor the occurrence of frost which has been shown to be detrimental to *R. ferrugineum* growth (Jonas et al., 2008; Choler, 2015). Following Carrer et al. (2019), one might also argue that warmer winter temperatures can increase snowpack density (Judson and Doesken, 2000), and thereby lead to longer snow persistence.

Before the 1990s, correlations between vessel sizes (MLA25) and growing season (May 8 – September 5) air temperature (r=0.801, p<0.05) were much stronger than between any climate parameter and the RW and CNo chronologies, whereas the correlation between winter (January 12 – March 19) precipitation totals and MLA25 (r=-0.684, p<0.05) was slightly higher with respect to the correlations with RW (r=-0.598, p<0.05) and CNo (r=-0.638, p<0.05) (**Figures 3**, **Figure 4**, **Table 2**). Conversely, correlations with late-summer air temperature in the year preceding ring formation (n–1) were weaker in the MLA25 chronology (r=0.528, p<0.05) compared to the RW chronology and a winter air temperature signal was almost absent (r= -0.397, p<0.05) prior to the 1990s (**Figure 3**, **Figure 4**, **Table 2**). Our results thus strongly support the hypothesis that air temperature in cold-limited environments primarily determines the water conduction capacity of xylem cells by influencing vessel lumen diameters (Kirdyanov et al., 2003; Fonti et al., 2013). In addition, the formation of wider vessels in years with warm air temperatures – during which vulnerability to freezing-induced xylem cavitation is reduced – could result from a shift in the trade-off between efficiency and safety reweighted toward efficiency (Tyree et al., 1994; Gorsuch et al., 2001; von Arx et al., 2012; Nielsen et al., 2017). The strong correlation between MLA25 and current (n) May-early September temperature (**Figure 3**, **Table 2**) can also be seen as the combined effect of two distinct signals, i.e. (i) accelerated snowmelt due to warm air temperatures in May and therefore an earlier start of the growing season (Francon et al., 2017, Francon et al., 2020) as well as (ii) vessel enlargement as a result of warm summer (JJA) air temperatures. In addition, the weak response of MLA25 to previous summer/fall air temperatures (n–1) tends to confirm the assumption of cell enlargement being more strongly influenced by direct insolation and air temperature than by the residual positive effects of warm conditions in the year preceding ring formation (Martin-Benito et al., 2013).

### 3.3 Fading climatic signal in cell chronology

By contrast, the same correlations between the different *R. ferrugineum* chronologies and air temperature decrease sharply when analyzed over the period 1990-2019 (**Figure 3**). Interestingly, although correlations with summer temperatures (June 10 – September 8, n) remain statistically significant (p<0.05) in the RW and CNO chronologies, they decrease markedly to 0.42 and 0.40, respectively. By contrast, in the case of the MLA25 chronology, relationships with air temperatures (n) are not significant anymore irrespective of the time window selected (i.e. any time window between 10 and 150 days) (**Figure 3**, **Table 2**). The winter air temperature and precipitation signals decrease equally in the three chronologies (**Figure 3**, **Figure 4**, **Table 2**) while correlations with summer (July 1 – July 21) precipitation totals (r = -0.6 for RW, -0.51 for CNo, and -0.55 for MLA25, p < 0.05, respectively) become significant during this period (**Figure 4**, **Table 2**).

This evolution of ring widths–climate relations observed at our study site somewhat echoes the “divergence problem”, first identified by Briffa et al. (1998). This phenomenon refers to the increasing loss of boreal forest growth to air temperature signals since the late 20^th^ century. Ever since, signs of divergence have been reported for various tree species growing at high-latitude (e.g., D’Arrigo et al., 2008) and high-altitude sites (e.g., Büntgen et al., 2008). The results reported in this study on *R. ferrugineum* are not only in line with work presented earlier on changes in tree growth, but also align nicely with recent studies reporting similar losses or shifts in climate sensitivity of shrubs and herbs to climate at local (e.g., Gamm et al., 2018 in Western Greenland; Weijers et al., 2018 in the central Norwegian Scandes; Dolezal et al., 2020 in the Low Tatras of Slovakia; Francon et al., 2020a; Francon et al., 2020b in the Northern French Alps) and regional (see Buchwal et al., 2020 in the Arctic; Francon et al., 2021 in the Alps) scales since the 1990s.

Yet, such a drastic loss of a climatic signal like the one documented in our study for cell lumen area (MLA25) has not hitherto been observed and represents a completely new level of signal loss in shrubs. The findings we report here are all the more unexpected as one major argument for the development of quantitative wood anatomy relies on the assumption that series of anatomical features would be far less influenced by external disturbances and more directly connected to climate parameters than RW, and that wood anatomical series should thus provide more stable relationships with climate (George and Esper, 2019; Björklund et al., 2020). In fact, Lehejček et al. (2017) have shown that cell wall thickness chronologies of *J. communis* shrubs exhibit stable correlations with summer air temperatures and the summer standardized precipitation evapotranspiration index (SPEI) and that the same correlations could not be observed in RW or other growth parameter series (including MLA). In line with Mérian and Lebourgeois (2011), one could therefore argue that the loss of climate signals since the 1990s could result from higher inter-individual variability. Yet, the strong increase of inter-series correlation (**Figure 2A**, **Table 1**, **S1**) observed after 1990 argues against this hypothesis in our case. Instead, we rather adhere to two alternative hypotheses – being of climatic and edaphic origins – to explain the evolution observed at our site.

In a context of global warming, one might think that the recent loss of vessel sensitivity to temperature could result from a reduction of vessel lumen area aimed at protecting the plant against more frequent summer drought and frost-induced cavitation (Tyree et al., 1994). Yet, the negative correlation that we observe between the MLA25 chronologies and summer precipitation since the 1990s and the location of the sampled individuals – at treeline, on a north-facing slope – leads us to reject the summer drought hypothesis. By contrast, and relying on results of a study from the French Alps using a locally calibrated meteorological model, specifically designed for the mountain environment, Francon et al. (2020) showed the detrimental effect of spring frost events on *R. ferrugineum* radial growth. Likewise, the sharp growth reductions observed in 2001 and 2005-2008 (**Figure 2B**) at our study site might indeed be attributed to frost and possibly add further evidence to the role of frost on MLA25 and the associated loss of MLA25 sensitivity to conventional climate parameters since c. 1990. Yet, in the absence of local meteorological data, this assumption lacks on-site validation.

In addition, further factors related to climate and/or morphological traits might have contributed to the signal loss observed in our study, including a phenological shift in time due to recent climate warming (e.g., Nagy et al., 2013; Ranjitkar et al., 2013; Prevéy et al., 2020; Rosbakh et al., 2021). Although differences in age/size within the population as well as a divergence in inter-individual resistance to frost (e.g., Marchand et al., 2020) could also be related to a climate signal loss, the strong inter-series correlation values observed in our study together with the common growth-reduction responses observed in all individuals independently of their age/size, tend to exclude the latter hypothesis.

A second hypothesis might be considered in which the loss of climate sensitivity of *R. ferrugineum* may have been exacerbated as a result of permafrost degradation in the Castelletto rock glacier. Although a thick layer of debris acts as a strong insulator and consequently dampens ice melting in rock glaciers, several studies report a marked warming of ice in rock glaciers and a related acceleration in rock glacier movements in the European Alps over the last decades (e.g., Hoelzle et al., 2001; Evatt et al., 2015; Pruessner, 2017; Anderson et al., 2018; Jones et al., 2019; Cusicanqui et al., 2021). At Castelletto, we cannot rule out the possibility that the succession of warm summers (**Figure S4**), amongst which the 2003 heatwave, may have reduced ice content and increased active layer depth sufficiently (Gruber et al., 2004; Åkerman and Johansson, 2008; Nowinski et al., 2010) to have repercussions on *R. ferrugineum* growth. Indeed, an increase in water and nutrient availability due to permafrost thaw is expected to favor resource acquisition, shrub growth (Iturrate-Garcia et al., 2020), and expansion (Limpens et al., 2021), at the expense of their resistance to stress (e.g., extreme climatic events, mechanical and hydraulic failure, pests), caused by a slower bark thickness and stem density production (Díaz et al., 2016; Iturrate-Garcia et al., 2020). In our study, the combined effects of the recent variation of site-specific edaphic conditions (i.e., permafrost degradation leading to changes in soil properties) along with climate warming may be larger than the impact exerted by a single-main driver (e.g., summer temperature) on shrub growth. This in turn could point to a far more complex response to interacting macro- and micro-environmental factors. Yet, in the absence of local permafrost data, this assumption cannot be validated with on-site data. Therefore, to better understand the anatomical and growth responses of shrubs to climate change in rock glacier environments characterized by complex interactions between rising air temperatures, permafrost degradation, and evolving soil properties, we recommend relying on monitored rock glaciers when assessing shrub growth in permafrost environments in future studies.

## 4 Conclusions

Our study is the first to provide insights into the anatomical and growth response of a ring-porous shrub species, *R. ferrugineum*, to climate warming in the Alps. The strong correlations computed between summer air temperatures and the mean lumen area (MLA25) chronology developed not only confirm the robustness of our approach but also underline the potential of shrub wood anatomy to track past climate variability at high elevation sites. Similarly, the winter precipitation signal detected in MLA25 is consistent with previous dendroecological studies showing negative effects of long-lasting snow cover on shrub radial growth in the Alps (Carrer et al., 2019; Francon et al., 2020a). At the same time, however, the strong decoupling of vessel size from climate since the 1990s echoes a divergence which has been reported previously for *R. ferrugineum* radial growth in the Alps (Francon et al., 2021). Yet, the amplitude of the divergence reported in our study is both unprecedented and unexpected as wood anatomy has hitherto been considered to provide much more stable relationships with climate parameters than ring width (Lehejček et al., 2017; Björklund et al., 2019). We offer several hypotheses to explain this loss of climate sensitivity, including a drastic evolution of the active layer of the rock glacier due to thawing permafrost (Åkerman and Johansson, 2008), an increased exposure of *R. ferrugineum* to late frost or winter drought (Neuner et al., 1999; Jonas et al., 2008) or a phenological shift in time due to recent climate warming. To test these hypotheses, we recommend future studies to focus on monitored rock glaciers where a more in-depth comparison of wood anatomical responses of shrubs would be possible on rock glaciers and adjacent ice-free areas.

## Data availability statement

The raw data supporting the conclusions of this article will be made available by the authors, without undue reservation.

## Author contributions

SP: Conceptualization, Data Collection - Preparation, Formal Analyses, Writing - Original Draft, Visualization LF: Conceptualization, Data Preparation, Formal Analyses, Writing - Review and Editing, Visualization CC: Conceptualization, Writing - Review and Editing, Supervision MS: Conceptualization, Writing - Review and Editing, Supervision LS: Data Preparation NC: Conceptualization, Review and Editing, Supervision. All authors contributed to the article and approved the submitted version.
