# Erratum: Vessels in a *Rhododendron ferrugineum* (L.) population do not trace temperature anymore at the alpine shrubline

**DOI:** 10.3389/fpls.2023.1170080

**Published:** 2023-03-06

**Authors:** Frontiers Production Office

**Affiliations:** Frontiers Media SA, Lausanne, Switzerland

**Keywords:** alpine shrub, dendroecology, wood anatomy, climate-growth relations, climatic signal loss

An omission to the funding section of the original article was made in error. The following sentence has been added: “Open access funding was provided by the University of Geneva”.

The original version of this article has been updated.

