# Supplementary material for: Vessels in a Rhododendron ferrugineum (L.) population do not trace temperature anymore at the alpine shrubline
Source: Front Plant Sci. 2023 Jan 12;13:1023384. doi: 10.3389/fpls.2022.1023384 (PMC9879627; doi:10.3389/fpls.2022.1023384)
Supplement: Supplementary file 1 [file DataSheet_1.pdf]

Vessels in a *Rhododendron ferrugineum* (L.) population do not trace temperature anymore at the alpine shrubline

SUPPLEMENTARY MATERIAL

Figure S1

Principal component analysis (Varimax PCA) performed to select a subsample of complementary variables, using the chronologies calculated from the detrended individual time series (RW= ring width, CNo= cell number, RA= ring area, MLA25= 25th percentile of lumen area distribution, CA95= 95th percentile of lumen area distribution, MLA= mean lumen area, Dh= hydraulically weighted mean vessel diameter, Ks= xylem-specific hydraulic conductivities).

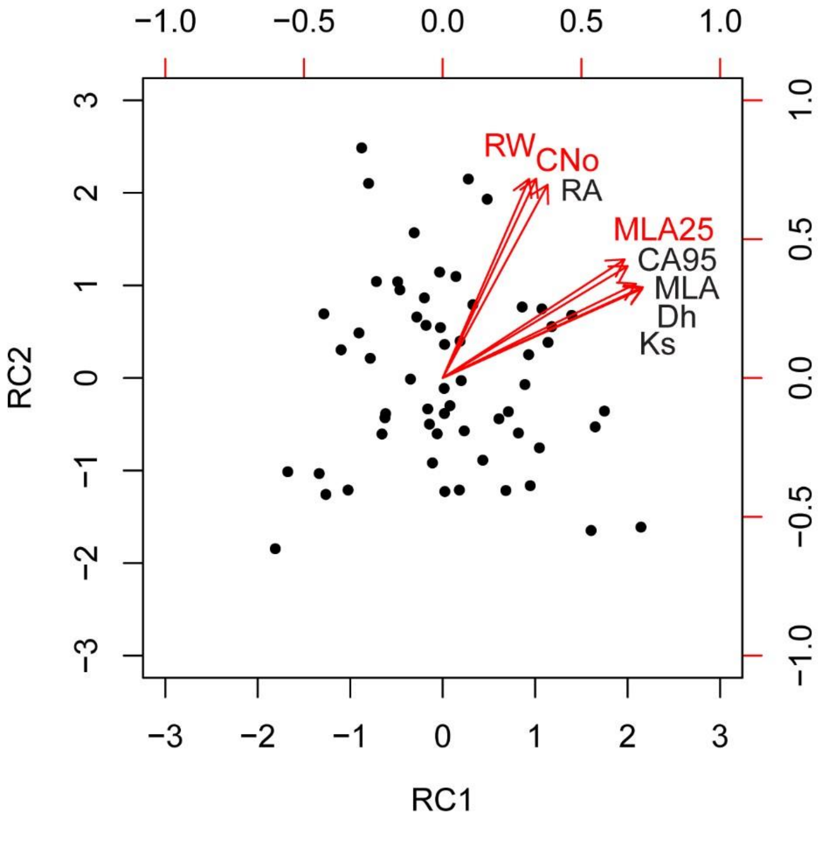

Figure S2

Monthly and seasonal bootstrapped correlation functions (BCF) using the Treeclim package in R. Monthly climatic data (red= temperature; blue= precipitation). The time period considered for the analyses included monthly variables from August of the year preceding the growth-ring formation (n − 1) to August of the year of actual ring formation (n). The analysis was computed over the entire investigated period (1960-2019) on the three selected parameters (RW=ring width, CNo=cell number, and MLA25= 25th percentiles of lumen area distribution).

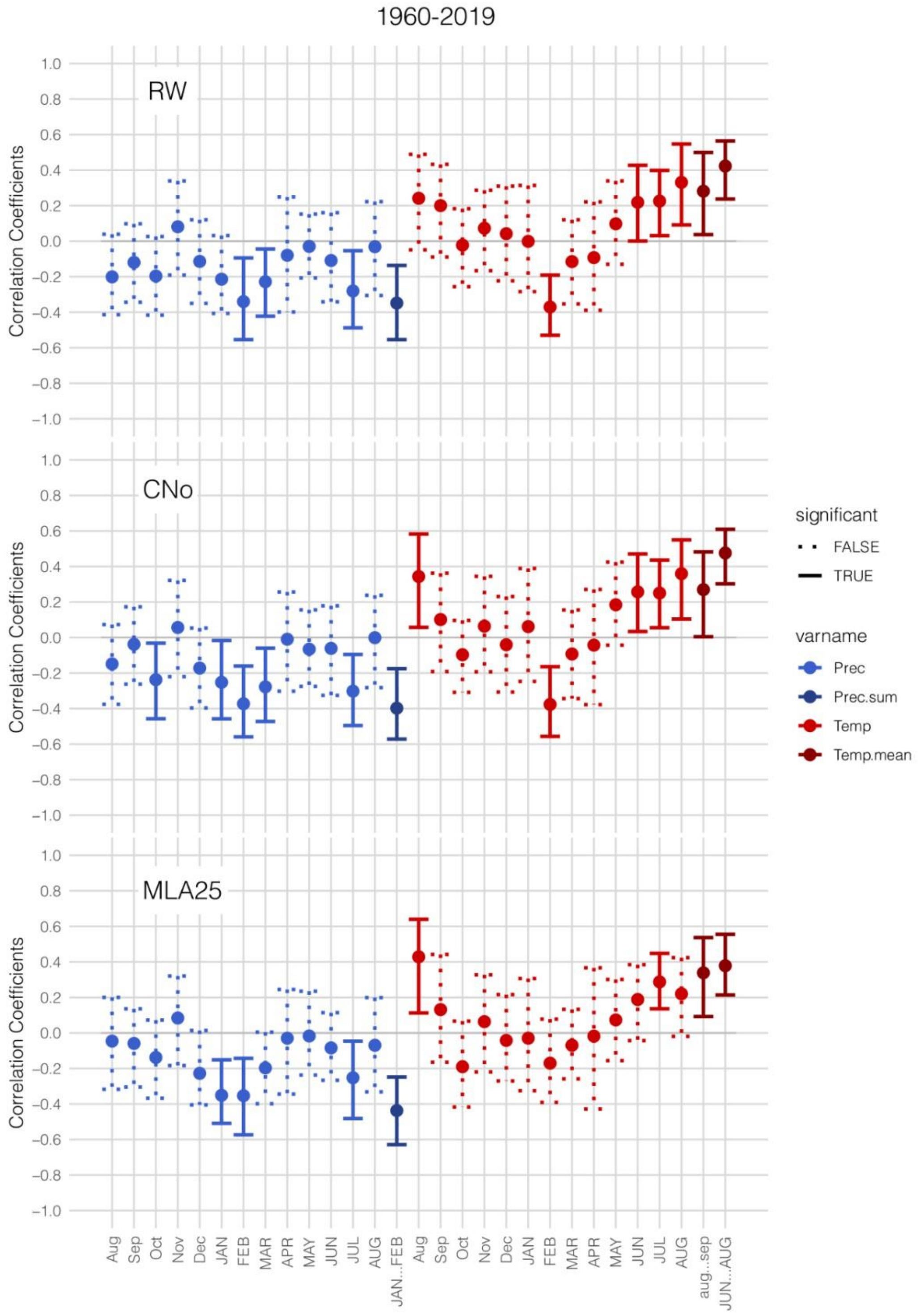

Figure S3

30-years moving windows correlation functions computed using the Treeclim package in the R package between the three selected chronologies (RW=ring width, CNo=cell number, and MLA25= 25th percentiles of lumen area distribution) and monthly and season climatic data (temperature and precipitation) for the entire investigated time period (1960-2019). The black frames show the two selected periods for each parameter. The asterisks indicate significant correlations ($P < 0.05$).

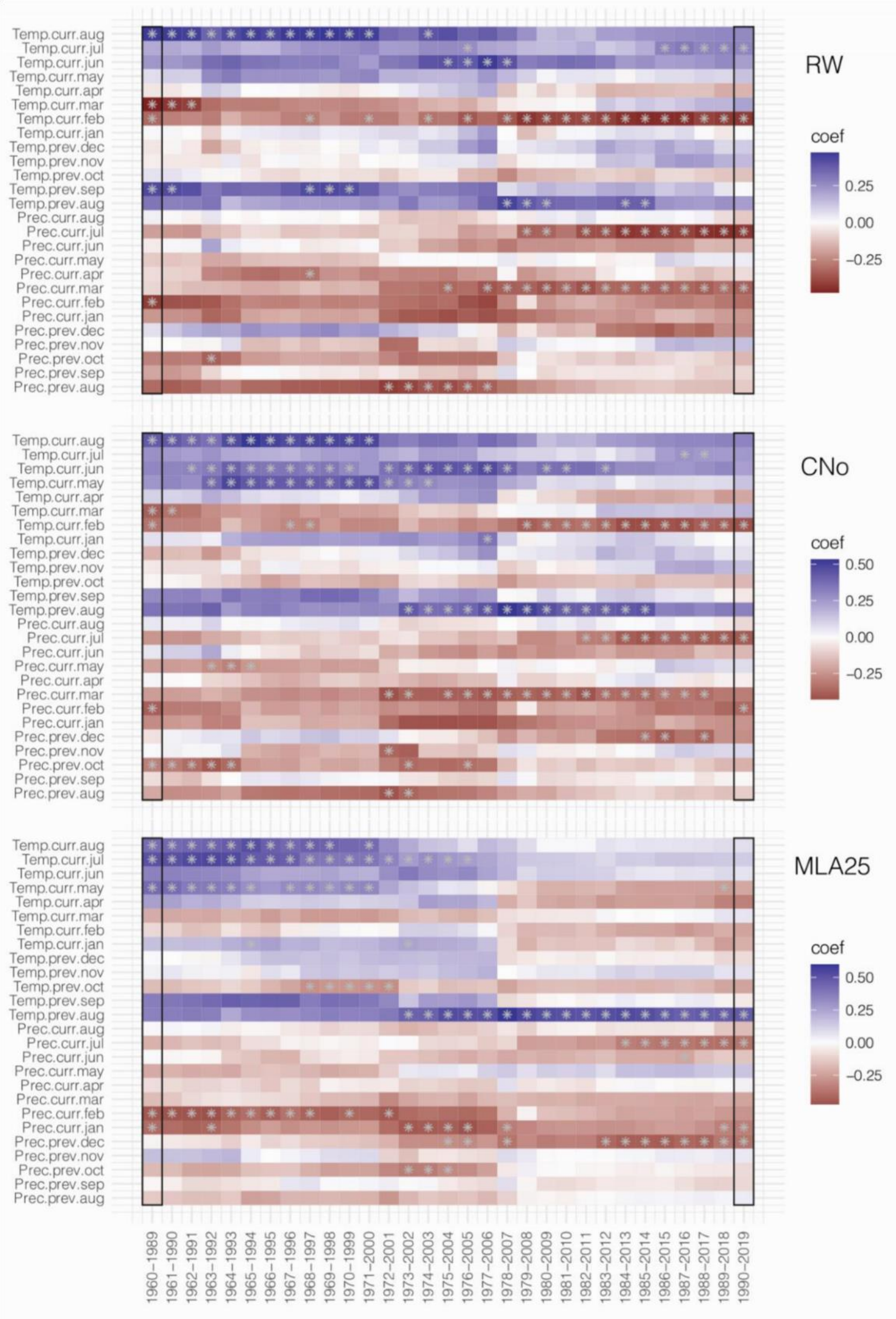

Figure S4

Summer (June, July, August) temperature (A) and precipitation (B) trends during the investigated periods (1960-1989 and 1990-2019). Data from Segl-Maria automatic weather station (46°26' N, 9°46' E, 1804 m asl). The dashed horizontal lines and the related values indicate the temperature (orange= 1960-1989, red= 1990-2019) and precipitation (light blue= 1960-1989, dark blue= 1990-2019) averages of each period.

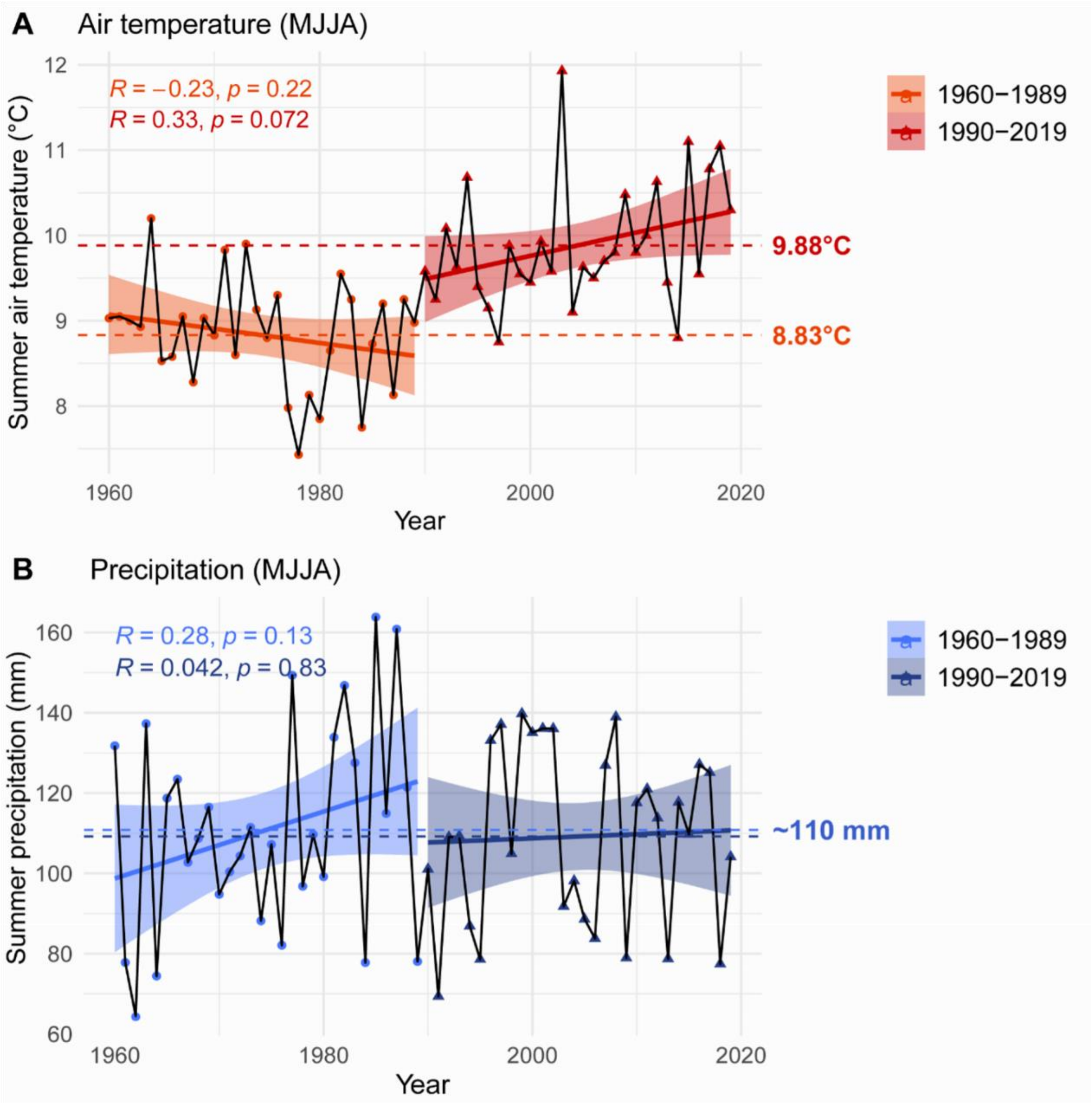

Table S1 Characteristics of *Rhododendron ferrugineum* chronologies of the three selected parameters (RW=ring width, CNo=cell number, and MLA25= 25th percentiles of lumen area distribution): mean annual size (± standard deviation) computed on raw chronologies and signal strength (rbar and SSS for both investigated periods: 1960-1989, 1990-2019) calculated after detrending within each period (Splined_30_0.5).

Wood parameters	Time period	Raw data		Splined_30_0.5	
		**Mean size**	**Unit**	**Rbar**	**EPS**
**RW**	1960-1989	0.121±0.041	mm	0.416	0.883
	1990-2019	0.140±0.058	mm	0.540	0.914
**CNo**	1960-1989	3.593±0.999	no.	0.376	0.808
	1990-2019	3.977±1.588	no.	0.596	0.930
**MLA25**	1960-1989	1.429±0.127	µm²	0.215	0.657
	1990-2019	1.585±0.156	µm²	0.449	0.880
